# Determination of Polycyclic Aromatic Hydrocarbons and Their Methylated Derivatives in Sewage Sludge from Northeastern China: Occurrence, Profiles and Toxicity Evaluation

**DOI:** 10.3390/molecules26092739

**Published:** 2021-05-06

**Authors:** Rashid Mohammed, Zi-Feng Zhang, Ze Kan, Chao Jiang, Li-Yan Liu, Wan-Li Ma, Wei-Wei Song, Anatoly Nikolaev, Yi-Fan Li

**Affiliations:** 1 International Joint Research Center for Persistent Toxic Substances (IJRC-PTS), State Key Laboratory of Urban Water Resource and Environment, Harbin Institute of Technology (HIT), Harbin 150090, China; m13045102069@163.com (R.M.); llyan7664@163.com (L.-Y.L.); mawanli002@163.com (W.-L.M.); weiweiwendysong@126.com (W.-W.S.); 2 International Joint Research Center for Arctic Environment and Ecosystem (IJRC-AEE), Polar Academy/School of Environment, Harbin Institute of Technology (HIT), Harbin 150090, China; 3 Heilongjiang Provincial Key Laboratory of Polar Environment and Ecosystem (HPKL-PEE), Harbin Institute of Technology (HIT), Harbin 150090, China; 4 Heilongjiang Institute of Labor Hygiene and Occupational Diseases, Harbin 150028, China; yuanban888@126.com (Z.K.); sey53969319@126.com (C.J.); 5 Institute of Natural Sciences, North-Eastern Federal University, 677000 Yakutsk, Russia; an.nikolaev@s-vfu.ru; 6 IJRC-PTS-NA, Toronto, ON M2N 6X9, Canada

**Keywords:** Me-PAHs and PAHs, risk assessment, sludge, source apportionment

## Abstract

This paper assesses the occurrence, distribution, source, and toxicity of polycyclic aromatic hydrocarbons (PAHs), and their methylated form (Me-PAHs) in sewage sludge from 10 WWTPs in Northeastern China was noted. The concentrations of ∑PAHs, ∑Me-PAHs ranged from 567 to 5040 and 48.1 to 479$\;ng.g^{-1}$dw, which is greater than the safety limit for sludge in agriculture in China. High and low molecular weight 4 and 2-ring PAHs and Me-PAHs in sludge were prevalent. The flux of sludge PAHs and Me-PAHs released from ten WWTPs, in Heilongjiang province, was calculated to be over 100 kg/year. Principal component analysis (PCA), diagnostic ratios and positive matrix factorization (PMF) determined a similar mixed pyrogenic and petrogenic source of sewage sludge. The average values of Benzo[a]pyrene was below the safe value of 600$\;ng.g^{-1}$ dependent on an incremental lifetime cancer risk ILCR of 10^−6^. Sludge is an important source for the transfer of pollutants into the environment, such as PAHs and Me-PAHs. Consequently, greater consideration should be given to its widespread occurrence.

## 1. Introduction

Polycyclic aromatic hydrocarbons represent a group of aromatic hydrocarbons with two or even more fused benzene rings, which were some of the main classes of organic hydrophobic pollutants. PAHs were recognized as the primary component responsible for the impacts of the organism [1]. Some PAHs have a great concern because of their carcinogenic effects, including BaA, BbF, BkF, BaP, IcdP, and BahA [2]. The International Agency for Research Cancer (IARC, 2010) has long recommended that benzo[a]pyrene a level 1 carcinogen, based on sufficient evidence in humans and animals [3]. Furthermore, PAHs are present in the environment; they have been discovered in fossil fuels, wildfires, natural vegetation, and volcanoes [4]. Methylated polycyclic aromatic hydrocarbons (Me-PAHs), one among PAHs derivative groups, are widely distributed in the environment, such as unsubstituted PAHs. Additionally, Me-PAHs such as methyl naphthalene (Me-Nap), methyl phenanthrene (Me-Phe), and methyl chrysin (Me-Chy) were recognized for industrial, petrogenic, or incomplete combustion sources [5]. However, (Me-PAHs) have been reported in WWTPs; their toxicities are sometimes higher than their corresponding PAHs [6]. Generally, PAHs and Me-PAHs in wastewater originate from industrial wastewater, domestic sewage, atmospheric precipitation, road runoff [7].

Sewage sludge is defined as a mixture of the residuals from WWTPs receiving urban wastewater or other wastewater of similar composition. In general, it is a liquid or a semi-liquid phase, with a substantial percentage varying from 0.25 to 12% by weight, depending on the operations and processes used [8]. The occurrence of PAHs in sludge was investigated since the 1970s in industrial countries such as the United States of America, the United Kingdom, and Canada [9]. PAHs and Me-PAHs in sludge through different pathways enter the environment; basically, it depends mostly on the sludge removal processes, land application after appropriate treatment is suggested approach of final sludge removal [10]. The differences in PAHs concentration in the sludge are assessed, basically depending on the geographical differences as well as the nature of the WWTPs processes [11]. Furthermore, change of soil PAHs concentration after addition of sludge the combined outcome of several mechanical, such as adsorption, desorption, bioformation, volatilization, photodegradation, leaching, and incorporation into the structure of the humic substance [12]. The organic pollutants contained in the sludge can stay in the soil for months to years because of their sorption to organic, mineral, and amorphous phases of soil and slow rates of biodegradation [13]. However, toxic compounds, such as PAHs, in the environment, released waste behaves as secondary contaminants. In addition, soil biota adversely affected by sewage sludge [4]. On the other side, sewage sludge could be used as fertilizer in the soil. Its significant impact is mainly on the crop yield by enhancing soil qualities through the enrichment with organic matter. Conversely, the adverse effects due to the presence of PAHs and other contaminants in sewage sludge has to be controlled [14]. In China, besides domestic, municipal wastewater, industrial wastewater treatments are being one production source of the incredible amount of sewage sludge due to the fast development, modernization, and industrialization [15], the quantity of the sewage sludge was very swiftly increasing from thirty million metric tons in 2012 to thirty-four million metric tons in 2015 [16]. Several previous studies have reported that the total content of PAHs in sludge varies between 4.0 and 50 mg/kg [17]. Another study from Beijing China, reported that the total PAHs concentration resulting from sewage sludge combustion averaged 6.103 mg/kg [18]. These days, the sludge issue is a tremendous challenge in China, so it is essential to manage, treat and dispose of sludge correctly to prevent severe environmental pollution [19]. Therefore, to the Chinese national standard (GB 4284–2018) was recently implemented specifying the maximum concentration of PAHs in treated sewage sludge for land application: 5 mg kg^−1^ and 6 mg kg^−1^ dry weight for Class A and Class, respectively [20]. Therefore, evaluates the quantity of sludge associated with PAHs and Me-PAHs from WWTPs is significantly related to sludge disposal regulations, toxicity estimation, and risk evaluation in sewage sludge. The objectives of the current research are: first, to investigate occurrence and profiles of PAHs and Me-PAHs in sewage sludge from 10 WWTPs in Heilongjiang province, China; second, to discover different potential sources collected from domestic and industrial sewage sludge WWTPs; third, to evaluate environmental toxicity of PAHs and Me-PAHs pollutants, which will provide valuable information according to the disposal regulation of sludge-amended soil and agriculture in Northeastern China.

## 2. Materials and Methods

### 2.1. Sample Collection

The sewage sludge samples were collected from 10 WWTPs along the Songhua River in the Northeast of China, Heilongjiang province. The detailed information of sampling sites was presented in Table S1. Ten sludge samples were collected starting from June, July until October. Six sampling sites were chosen in the most populated and industrial area of Harbin (W5, W6, W7, W8, W9, and W10), two sampling sites from Jiamusi Eastern and Jiamusi Western (W3 and W4) and two sampling sites from Qiqihar and Mudanjiang (W1 and W2). All samples were gathered from an anoxic/oxic (A/O) biological tank and sludge dewatering tank from WWTP. All samples were gathered (i) tacked and saved in aluminium containers, (ii) then freeze-dried, and (iii) finally stored in darkness under −20 °C. All samples were transmitted in the International Joint Research Center for Persistent Toxic Pollutants (IJRC-PTS), Harbin Institute of Technology (HIT), Harbin, China.

### 2.2. Chemicals and Reagents

High-performance liquid chromatography (HPLC) exhibiting grade-quality was used as a solvent in the experiments described in the present study. Dichloromethane (DCM), methanol (MeOH). Additionally, toluene was obtained from Fisher Scientific, Fair Lawn-New Jersey-USA. A Milli-Q system, Millipore-Billerica-MA, was used to prepare Pure reagent water (>18 MΩ-cm R). 

Each of the PAHs mixture standards was obtained from the Accuse Standard (New Haven, CT, USA). The 98% pure deuterium-labelled 16 PAHs mixture standards were purchased from AccuStandard. 2-Methylnaphthalene-d_10_, 1-Methylnaphthalene-d_10_, 9-Methylanthracene-d_12_, 2,6-Dimethylnaphthalene-d_12_ were purchased from Chiron (Norway).

### 2.3. Sample Pre-Treatment and Instrumental Analysis

Sludge samples were extracted by ultrasonic extraction. We carried out the following steps sample for pre-treatment instrumental analysis: (i) 0.5 g of sludge sample, 80% moisture content grinded with anhydrous Na_2_SO_4,_ (ii) 25 mL mixture solvent of MeOH-DCM (1:1 *v/v*) were added, (iii) then extracted for 30 min, (iv) and 5 min was centrifuged at 3000 rpm, (v) after that extraction has been repeated twice, (vi) and the extract was gathered into a flask, then (vii) approximately 1 mL of extract was concentrated, (viii) and re-dissolved to 500 mL purified water, finally (ix) the solution has been extracted by the solid-phase extract. HLB cartridges to ($500\;\mathrm{mg}.6{\;\mathrm{cc}}^{-1}$) (Water Milford, MA, USA) were noticed of DCM with 5 mL and 5 mL of MeOH, accompanied by ultrapure water (5 mL) at a rate of approximately 1 mL min-1. After that, water samples (1 L) were loaded at a rate of about 5 $mL.min^{-1}$. Then, drying for 60 min with a gentle stream of N2, the SPE cartridges were fully clarified from the sorbent as follows: (i) into 15 mL tubes with 7 mL DCM and (ii) into 7 mL MeOH at a flow rate of 1 mL/min. A gentle stream of N2, around 1 mL, was used to extract the extracts, and the solvent was changed to 1 mL with toluene until being shifted to 1.5 mL. For sediment, ultrasonic extraction had been used. The sediment sample was dried and homogenized, whereas 3–5 g of MeOH-DCM (1:1 *v/v*) mixture solvent had been ultrasonically for 20 min extracted and for 5 min centrifuged at 3000 rpm, the supernatant then was picked, and the extraction was repeated several times. 

The detection of PAHs and Me-PAHs was performed by use of Agilent 7890A-7000B gas chromatography-tandem triple-quadrupole mass spectrometry applied to an EI ion source (GC-EI-MS/MS) and (MRM) chromatogram. An agilent 19091J-433E (30 m × 250 µm × 0.25 µm) HP-5MS chromatographic column was employed in GC. All the parameters of transitions collision energy and retention time were listed in (Table S2) and (Figures S1 and S2).

### 2.4. Quality Assurance/Quality Control (QA/QC)

All of the data were established, including quality assurance (QA) as well as quality control (QC). A blank procedural and a matrix spike (20 ng/g dry weight with sludge samples and 100 ng/g for samples), and to verify the contaminants, an extra matrix spike was accurately checked, peak identification and measurement in each batch of 12 samples analyzed. Unchanging level of internal standard (100 ng/mL) with a sequence of injections of objective compounds at different concentrations was attained to figure out the system of a linear range. If each of the sample extracts reach the range, it would be diluted appropriately to get the reaction within the calibration range.

### 2.5. Data Analysis

#### 2.5.1. Positive Matrix Factorization and Statistical Analysis 

The statistical analyses were performed using SPSS Statistic V25 software. The diagnostic ratio approach was utilized to determine potential sources of PAHs and Me-PAHs in several functional areas, and principal component analysis (PCA) was also chosen for discovering possible sources. Pearson correlation analysis was applied to check out the relationships between concentrations of PAHs and Me-PAHs in sewage sludge. The US EPA Positive matrix factorization (PMF) model V.5 software was utilized. PMF is a multivariate factor analysis tool that decomposes the data matrix into 2 matrices: factor contributions and factor profiles with a residual matrix [21]. The weighted sum of squares (Q) value is the difference between the data set input and PMF output [22], where the objective function (Q) is minimized in the PMF solution, as displayed in Equation (1).

(1) $$Q=\overset{n}{\underset{i=1}{\sum }}\overset{m}{\underset{j=1}{\sum }}{\left(\frac{X_{ij}-\sum_{k=0}^{p}g_{ik}f_{kj}}{U_{ij}}\right)}^{2}$$

whereas (f_kj_) profiles a species of each source, (g_ik_) is mass contributed by each factor to each sample, (p) is the number of factors, (i) is measured in the sample, and (U_ij_) is the uncertainty estimate of the source (j). The uncertainty (U_nc_) matrix values were calculated based on the PAHs method detection limits utilizing Equations (2) and (3) [23], as follows:

(2) $$U_{\mathrm{nc}}=\frac{5}{6}\times \mathrm{MDL}$$

(3) $$U_{\mathrm{nc}}=\sqrt{{\left(\mathrm{Error}\;\mathrm{Fraction}\;\times \mathrm{concentration}\right)}^{2}+{\left(0.5\times \mathrm{MDL}\right)}^{2}}$$

#### 2.5.2. Health Risk Assessment

The potential cancer risk for PAHs was calculated in Equation (4) by multiplying the concentration of each compound by its corresponding TEF values. The total BaP equivalent concentrations (BaPeq) were calculated from the individual PAHs concentrations in each sample (Ci) and the toxicity equivalency factor (TEF) of target compounds from the flowing equation:

(4) $$\sum \mathrm{BaPeq}=\sum \left(\mathrm{Ci}\;\times \mathrm{TEFi}\right)$$

where BaPeq is the carcinogenic potency of a congener evaluated based on BaP-equivalent concentration. TEF is the toxic equivalent factor provided [24]. 

#### 2.5.3. Fluxes Calculations of PAHs and Me-PAHs

Daily and annual fluxes of PAHs and Me-PAHs discharged from WWTP sewage sludge were calculated using the following equation:

(5) $$F\;=\;C\;\times \;M\;\times \left(1-0.8\right)$$

where © represents the concentration of ∑Me-PAHs and ∑PAHs in the sludge $ng.g^{-1}$dw, (0.8) defines as the evaluated fraction of water in the sludge, and (M) indicates the quantity of sludge from the wastewater [16].

## 3. Results and Discussion

### 3.1. Occurrence of PAHs and Me-PAHs in Sludge

**Figure 1:**
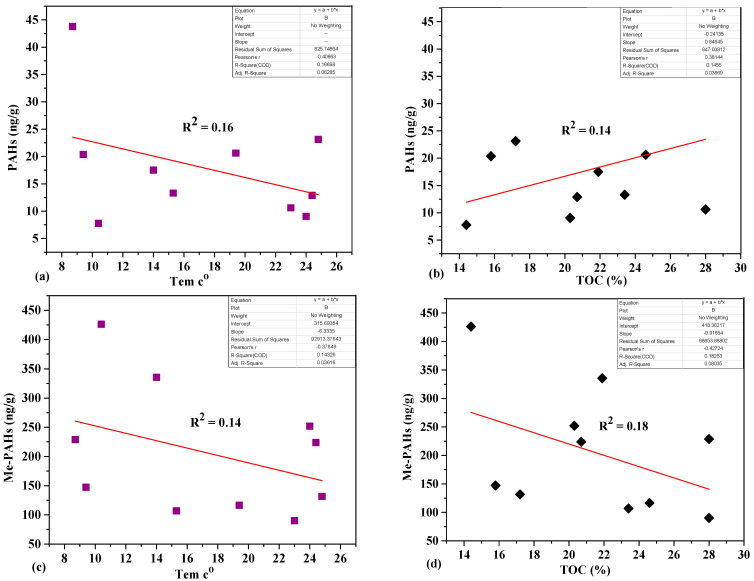
Correlation analysis between TOC, Temperature versus PAHs (**a**, **b**) and Me-PAHs (**c**, **d**) in sludge.

The concentrations of Me-PAHs and PAHs in sludge samples were summarized in Table 1. The concentrations of ∑PAHs varied from 567 to 5040 $ng.g^{-1}\;dw$, with an average 2030 ± 1340 $ng.g^{-1}$ dw. ∑Me-PAHs varied from 48 to 479 $ng.g^{-1}$, with an average of 205 ± 139 $ng.g^{-1}dw$. Moreover, the concentrations in three sites (W9, W2, and W5), located in two large urban cities (Mudanjiang and Harbin), which were the very significant industrial centers along the Songhua River, were up to 2500 ng/g dw. At the same time, the lowest value was discovered in site W6 (625 $ng.g^{-1}\;dw$), which was situated in a relatively low-density urban area. The levels of PAHs were relatively lower at the less populated sampling sites than high populated sites, which indicated the exposure depend directly on human activity and populated area; vehicle exhaust was generally regarded as the essential pollution sources in the metropolitan region of China due to increasing vehicle usage and opposed atmospheric conditions. Appropriate composition modifications, domestic and industrial, could explain why the PAHs and Me-PAHs composition of sludge is different from one site to another. The concentrations of ∑PAHs_carc_ seven carcinogenic (dibenzo[a,h]anthracene, benzo[a]pyrene, indeno[1,2,3-cd]pyrene, benzo[a]anthracene, benzo[k]fluoranthene, chrysene, and benzo[b]fluoranthene), displayed in (Table 1). The seven PAHs carcinogenic values vary from 213 to 1712 $ng.g^{-1}$ dw, and the greatest concentration was detected in the sludge from site W5, which is located in Harbin city, the most populated and old industrial city [25]. Whereas the levels of ∑PAHs_carc_ in this research were greater than five times than in Guangzhou, China, which was 201 to 308 $ng.g^{-1}$ dw [26], and nine time than in Tunisia, which varied from 16.14 to 1366 $ng.g^{-1}$ [14]. The outcome proved that the sludge was predominantly of urban origin in this research; this explains the higher PAHs content, which may contaminate sludge of industrial origin. Generally, PAHs concentration depends on the source position, including such industrialized areas or very highly populated regions [27]. In addition, 16 PAHs were lower in this study than in Zhuhai, China, which was between 53 and 25000 [9]. The characteristics of Me-PAHs, as well as PAHs, have been further investigated utilizing the statistical method, in particular, the correlation analysis (Figure S3). There was a significant positive weak correlation appeared between ∑Me-PAHs and ∑PAHs was noticed among the sampling sites, suggesting that similar emission sources were identified for these two groups (R^2^ = 0.02, *p* <0.01). Moreover, organic carbon and temperature were detected during the data measurement to explore their influence on the concentration of PAHs and Me-PAHs. The temperature varied from 8.7 to 24.8 °C, and the plot was comparing temperature and PAHs; Me-PAHs are displayed in Figure 1. Weak influences of temperature between PAHs, Me-PAHs and temperature were observed (R^2^ = 0.16, *p* < 0.01) as well as (R^2^ = 0.14, *p* < 0.01). Temperature is the main factor impacting the level of PAHs in sludge; previously, research stated that with an increase in temperature and increased PAHs removal rate in sludge. Meanwhile, the weak correlation observed with temperature suggested that the adsorption of PAHs increases at very low temperatures [28]. Moreover, as shown in Figure 1, positive weak linear correlations between the TOC and concentration of PAHs, Me-PAHs were also observed (R^2^ = 0.14, *p* < 0.01) as well as (R^2^ = 0.18, *p* < 0.01), indicating that TOC had a significant weak influence on PAH’s and Me-PAH’s distribution in sludge. A similar result showed that total organic carbon is one of the most important factors in detecting PAH sorption and immobilization [4].

### 3.2. Composition Profile

**Figure 2:**
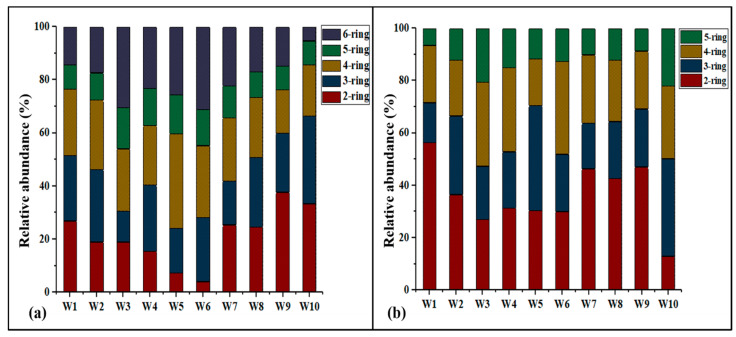
Average composition profiles of PAHs (**a**) and Me-PAHs (**b**) in sludge from 10 WWTPs.

The composition profiles of individual and ring-numbers of PAHs and Me-PAHs in sludge over the sampling sites are presented in (Figure 2 and Figure S3). It was shown that the most abundant PAHs were Phe (27%) in W10, followed by Nap (24%) in W9, and Fluo (16%) in W5, based on overall average samples. The same results were observed that Phe was Principally generated from fuel oil, diesel, gasoline, particularly petroleum products. The higher concentrations of Nap and Phe could, therefore, be attributed to industrial wastewaters [29]. Other research stated that low molecular weight compounds (e.g., Phe, NaP) discovered in this research was found in manufacturing areas as well as wastewater from household [14]. For Me-PAHs, the most abundant were 9-MANT (20%) W10, followed by 2-MNAP (16%), W9, and 5, 8-DMBcPH (11%) W6, as shown in Figure S3. Additionally, researchers reported benzene ring numbers were used to determine PAH physical and chemical characteristics [4]. According to the number of benzene rings which given PAHs contains, the Me-PAHs and PAHs were classified into (6-ring, 5-ring, 4-ring, 3-ring, and 2-ring) Figure 2. We found the PAHs homologs in sludge, rings number HMW PAHs (4-ring) were much dominated accounted for (42.5%) at site W5, followed by 3-ring (41.1%) at site W10, 2-ring (25.6%) at site W9, 5-ring (20.8) at site W3, and 6-ring (20.1%) at site W3, respectively, Figure 2. For Me-PAHs, low molecular weight PAHs (2-ring) were much dominated accounted for (55%) at site W1, followed by 3-ring (46.9%) at site W10, 4-ring (42.9%) at site W6, and 5-ring (3.2%), at site W10. Generally, the PAHs and Me-PAHs of sewage sludge in this research were dominated by HMW and LMW 4 and 2-ring; HMW PAHs occupied higher percentages; his might result in very highly hydrophobic characteristics of high molecular weight PAHs [3], whereas low molecular weight Me-PAHs may characterize by higher resistance to microbial decomposition and greater susceptibility to biological degradation [4]. This was in contrast with the previous finding as follows: sewage sludge from Tunisia was reported to be dominated by 4-ring [14]; similarly, in sewage sludge from Korea, high molecular weight 4-ring such as pyrene was the most abundant [30]. Four-ring PAHs were reported to be the most abundant compounds and accounted for 86% of ∑PAHs from Guangdong Province, China [31], and four-ring PAHs were prevalent in sewage sludge found in southwestern Taiwan, China [11].

### 3.3. PAHs and Me-PAHs in Sludge Worldwide

The comparison of the PAHs and Me-PAHs mean concentrations in sludge discovered from other researches around the world was shown in Table 2. The mean concentration of PAHs in this study was 2030 $ng.g^{-1}$dw, much greater than four sludge sites in Taiwan China (750 ng/g dw) [11], twelves sewage sludge from Beijing, China (1551 ng/g dw) [29], nineteen sewage sludge from Guangdong Province China (1276 ng/g dw) [7], but lower than the level in four sewage sludge from Harbin, Northeast China (8200 ng/g) [32], six sludge from Guangdong, China (3466 ng/g dw) [33], six Wastewater sludge from Korea (10,400 ng/g dw) [30], nineteen sludge from Madrid (5118 ng/g dw) [34], nine sewage sludge from northern and central Tunisia (11,216 ng/g dw) [14], and eleven sewage sludge from the mainland and Hong Kong, China (30,000 ng/g dw) [9]. However, the level in this study was very close to three sewage sludge from Paris (2518 ng/g dw) [35]. The European Union has proposed a limited fixed value of ten PAHs of 6000 $ng.g^{-1}$ for the implementation of sludge to agricultural fertilizer that includes Flua, BaP, IcdP, Ace, BghiP, BkF, Pyr, Phe, Flu, and BbF [29]. In our study, the values of 10 PAHs did not exceed the maximum limit. Therefore, these outcomes were at relatively low risk related to the European Union, with their agricultural land use. Nevertheless, corresponding to the disposal of sludge used as agricultural in China, from municipal wastewater treatment plants (5000 ng/g dw) [16], the concentration of ∑PAHs in this research was greater than the upper safety limit, with a comparatively high risk.

### 3.4. The PAHs and Me-PAHs Loads from Sewage Sludge Discharged from 10 WWTPs

Production of sludge in China was growing at an annual average rate of (13%), which was estimated at over 3000 tons of sludge in 2013 [36]. The estimated fluxes from the 10 WWTPs are presented in Table S4. The PAHs and Me-PAHs daily fluxes in the ten sewage sludge sites were ranged from 18.8 to 300 g/d and from 90 to 425 g/d, respectively. The annual quantity of flux varied from 6.99 to 111 kg/year and 33.3 to 157 Kg/year, respectively. Among sampling sites, W5 was dominated in both PAHs and Me-PAHs. The individual flux decreased in the followed orders: Fluo ˃ Phe ˃ Pyr ˃ BbF, and 9-MANT ˃ 2-MNAP ˃ 7,9-MBaA ˃ 1,6-DMNAP, respectively. The total flux in this study was much higher than the flux calculated from five sludge in Paris, France, which ranged from 0.53 to 316 g/d [37]. Moreover, more incredible than the quantity of released sludge from 15 WWTP in the Shanxi province ranged from 0.2 to 22.7 $kg\cdot year^{-1}$ [16]. Regarding the evaluation of the daily and yearly quantity of PAHs and Me-PAHs fluxes, sludge displayed the highest contamination level from the industrial and domestic areas in Northeast China compared to other quantities of flux worldwide.

### 3.5. Source Appointment

#### 3.5.1. Principal Component Analysis

The principal component analysis was utilized to recognize sources of PAHs and Me-PAHs by analyzing the principal components (PCs) extracted with total accumulated loading accounting for more than 90% of the variance. As presented in (Table S5), the two principal components were extracted for PAHs: PC1 and PC2 explained 67% and 22% of the total variance (89%). As shown in Table S5a, PC1 (67%) is mainly associated with high molecular weight PAHs, like Fluo, BKF, BaP, BbF, BaA, Pyr, IcdP, and BghiP, reported that highly loaded on BaP, BaA, BghiP, and IndP were the signals of exhaust emissions from gasoline-powered vehicles, and IcdP was related to gasoline, vehicle emissions [38]. Therefore, it was suggested that PC 1 could be considered as characteristic gasoline exhaust emissions. PC2 (22%) was highly associated with low molecular weight, Flu, Ace, NaP, Acy, and Phe. Oil and fuel spills, as well as incomplete combustion of oils and fuels, may produce these compounds [39]. For Me-PAHs, three principal components were extracted, PC1, PC 2, and PC 3 explained 58.8%, 22%, and 4.37% of the total variance (85.1%). As presented in (Table S5b), PC1 (58%) associated with 1-MPYR, 1,2-MBaA, 1-MPHE, 1-MANT, and 3,6-DMPHE. Several kinds of research have connected high loadings of MPHE to either unburned petroleum from vehicles [40]. Therefore, PC1 was related to coal and wood combustion. PC 2 (22%) was more influenced by 1,3-DMNAP, 1,4-, DMNAP, 1,2-DMNAP, and 1-MNAP, methylated derivatives of LMW NAP, it is derived from the combustion of wood, coal briquette, and charcoal [39]. PC3 (4.37%) was related to 9,10-DMA, 2-MNAP, 9-MMHEN, and 1-MNAP reported that the abundance of 2-MNAP in settled dust of kitchens with wood-burning stoves [39].

#### 3.5.2. Positive matrix Factorization

**Figure 3:**
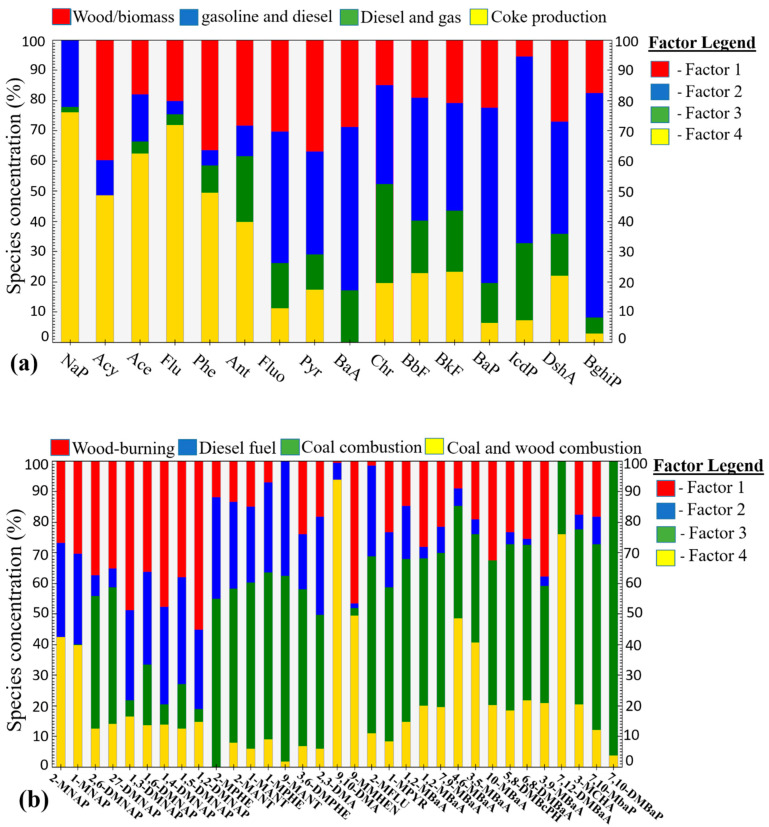
Fingerprints of positive matrix factorization (PMF) factors in each of the individual PAHs (**a**) and Me-PAHs(**b**).

Model PMF is a powerful and reliable method for solving various sources of pollutants [22]. It quantitatively assesses the contribution of PAHs contamination [23]. The sources of PAHs and Me-PAHs in sludge for this research were figured out using PMF. Four factors were obtained corresponding to PMF analyses (Figure 3). The first factor had a higher loading for Acy (40%), Phe (36%), Pyr (37%), and Flu (30%), Phe was a significant indicator of wood combustion, and Pyr and Flu were related to biomass burning; therefore, this factor represented a wood/biomass combustion source [41]. The second factor had high loading for high molecular weight BghiP (75%), IcdP (63%), and BaP (58%), this factor could be assigned to gasoline and diesel fuel, the same profile was reported in Brazil, consequences of heavy-duty vehicles [21]. The third factor had high loading for Chr (35%), IcdP (28%), BkF (20%), and BaA (16%), Chr and BaA were indicative of both diesel and natural gas combustion, BkF was the typical pollutant of diesel emissions; therefore, the third factor could be assigned of diesel and natural gas combustion [42,43]. The fourth factor had high loading for low molecular weight NaP (76%), Flu (72%), Ace (64%), and Phe (50%), which indicated a typical tracer of coke production [44]. Me-PAHs’ first factor had a high loading for 1,2-DMNAP (56%), 1,3-DMNAP (48%), 1,4-DMNAP (46%), and 3,9-MBaA (38%), it reported that the abundance of Me-NaP related to settled dust of kitchens with wood-burning stoves. Consequently, the first-factor source was wood-burning stoves [39]. Factor 2 showed abundant compounds of 9-MANT (38%), 1-MPHE (34%) and 3-DMA (30%), have reported that 2-MANT was one of the most prevalent PAHs compounds from vehicles and diesel fuel affect the environment [40], consequently, assigned this factor to diesel fuel vehicles. Factor 3 showed high loading of 7,10-DMBaP (70%), 7,10-MBaP (60%), 2-MFLU (58%) and 2-MPHE (54%); it reported that coal combustion might be mainly responsible for a high level of BaP concentrations, and coke production could be then utilized as indicative of this compound [2]; therefore, Factor 3 could be attributed to coal combustion. Factor 4 showed abundant compound of 9,10-DMA (94%), 7,12-DMBaA (76%) and 4,6-MBaA (47%); this profile is similar to methylated derivatives in northern Vietnam, high loading of MBaA indicating coal and wood combustion [39].

#### 3.5.3. Source Apportionment by Diagnostic Ratios

**Figure 4:**
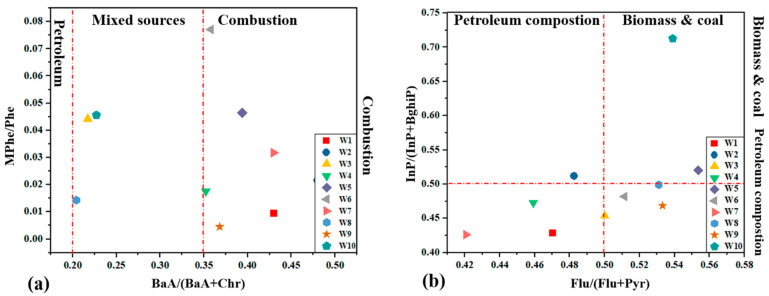
Plots ratios of BaA/ (BaA + Chr) Versus MPhe/Phe (**a**) and Flu/(Flu+Pyr) Versus InP/(InP + BghiP) (**b**).

Several concentration ratios of various parent PAHs compounds are commonly used to assess the sources of PAHs [27]. In the present study, the following diagnostic ratios were selected to assess PAHs and Me-PAHs source: BaA/(BaA + Chr) vs. (MPhe/Phe) and Flu/(Flu +Pyr) vs. InP/(InP + BghiP) (Figure 4). Since (PAHs) are released into the environment from a variety of sources, and their profiles can change due to their reactions, these ratios must be assessed with caution [21]. The result indicated that ratios of BaA/(BaA + Chr) vs. (MPhe/Phe) were ranged from 0.20 to 0.35, 0.35 to 0.50, and 0.00 to 0.008, respectively, indicated that dominated of pyrogenic origin was a primary source in the sewage sludge (Figure 4a), and diagnostic ratios of Flu/(Flu +Pyr) vs. InP/(InP + BghiP) were varied from 0.42 to 0.50, 0.50 to 0.58, 0.40 to 0.50 and 0.50 to 0.75, respectively, the PAHs at second group sites, as shown in (Figure 4b) were the highly petrogenic source in the sewage sludge. According to diagnostic ratios, it was proved that the sources of PAHs and Me-PAHs in sewage sludge were pyrogenic and petrogenic.

### 3.6. Toxicity Evaluation and Risk Assessment in Sewage Sludge

To evaluate the PAHs carcinogenic potency in sludge from 10 WWTPs, (BaPeq) benzo [a] pyrene equivalent concentrations were estimated by (TEFs) benzo [a] pyrene toxic equivalency factors. The U.S. EPA has suggested seven carcinogenic Polycyclic aromatic hydrocarbons, including (B(a)P, Chr, IcdP, B(a)A, BghiP, B(k)F, and B(b)F). The BaPeq concentrations of ∑16 PAHs in sludge were dominant in W5 with a total of 406 and meant concentration 25 ng/g, followed by W3, with a total of 197 and mean concentration 12.3 ng/g and W2 with a total 185 and mean concentration 11.6 ng/g, respectively. The PAH carcinogenic potencies, as well as toxic equivalency factors which are detected in sewage sludge, were displayed in Table 3, individual PAHs, the concentration of (BaP_eq_) toxic equivalency factor, values arranged in the following descending order: BaP ˃ DahA ˃ BbF ˃ BaA in all the 10 WWTPs sewage sludge, which were more abundant than other compounds. Sludge used in France as agricultural fertilizer is only allowed when: BaP, BbF, and Flu are below 2000 $ng.g^{-1}$, 2500 $ng.g^{-1}$, 5000 $ng.g^{-1}$dw, respectively, regarding the USA legislation is 4600 $ng.g^{-1}$dw, calculated as the sum of (Ant, Chr, DahA, BkF, IcdP BaP, BkF, and BaA), but BaP concentration was not allowed to be greater than 1000 $ng.g^{-1}$ [32]. In the Canadian soil quality guideline, dependent on (incremental lifetime cancer risk ILCR of 10^−6^), a safe benzo[a]pyrene equivalent value is 600 $ng.g^{-1}$, for seven carcinogenic polycyclic aromatic hydrocarbons, such as BbF, Chr, BaP, BkF, InP, DBA, and BaA [31], while the concentrations of benzo[a]pyrene equivalent to seven carcinogenic PAHs in sewage sludge among 10 WWTP in this study ranged from 52.3 to 399 $ng.g^{-1}$, with a mean concentration of 144 $ng.g^{-1}$ dw. The result proved that the concentrations in 10 WWTP sludge samples were below the safe value of 600$\;ng.g^{-1}$ (dependent on ILCR of 10^−6^).

## 4. Conclusion

In this research, the level of PAHs and Me-PAHs were investigated in sewage sludge. The most abundant PAHs compounds in sludge were Phenanthrene and Naphthalene, while Me-PAHs were 9-Methylanthracene and 2-Methylnaphthalene. The flux of sludge discharged from the 10 WWTPs, were estimated to be greater than 100 $kg\cdot year^{-1}$. The sources were analyzed by positive matrix factorization, principal component analysis, and diagnostic ratios, were identified similar source, analytical results of PMF explained 4 Factor, PAHs source includes, gasoline and diesel fuel (35%), coke production (33%), wood and biomass (19%) and average diesel and natural gas combustion, fore Me-PAHs, wood combustion (34%), coke production (28%) wood-burning stoves (22%) and average diesel fuel vehicle (16%). Temperature and total organic carbon were reported to have significant weak PAHs and Me-PAHs influencing distribution in sludge. The occurrence of PAHs in sludge confirms that there is a high risk for sludge applied to agriculture, according to China’s safety limit. Sludge acts as an important source for the transfer of pollutants such as PAHs and Me-PAHs into the environment; therefore, greater attention should be paid to its occurrence and distribution.

## Figures and Tables

**Table 1:** Concentrations of PAHs and Me-PAHs in sludge $\left(ng/g\;dw\right)$ from 10 WWTPs in Northeast China.

Compounds	W1	W2	W3	W4	W5	W6	W7	W8	W9	W10
∑PAHs	2040	2790	1950	1150	4410	625	1410	1340	2610	1940
∑Me-PAHs	106	252	224	132	425	228	147	116	90.0	335
∑PAHs-carc	460	705	775	369	1710	213	419	386	610	396
∑LMW PAHs	984	1250	547	469	1080	199	542	656	1450	1200
∑HMW PAHs	1060	1540	1400	686	3330	426	873	693	1160	743
∑LMW/HMW	0.92	0.81	0.38	0.68	0.32	0.46	0.62	0.94	1.25	1.61
∑LMW Me-PAHs	77.9	181	127	76.7	323	127	98.5	81.7	65.3	203
∑HMW Me-PAHs	28.9	70.4	96.9	54.8	102	102	48.7	34.6	24.6	132
∑LMW/HMW	2.69	2.57	1.31	1.40	3.17	1.23	2.02	2.36	2.64	1.53

W1 to W10, represent wastewater treatment plant sites. ∑LMW/HMW, Low molecular weight PAHs/High molecular weight PAHs =∑2–3rings/∑4–6rings. ∑PAHs-carc, Total concentration of potentially carcinogenic PAHs (BaA, Chr, BbF, BkF, BaP, InP and DahA).

**Table 2:** Comparison of average concentrations of PAHs ($ng.g^{-1}$ ) in sludge around the world.

Locations	No. of WWTPs	Sludge Types	Concentration (ng/g)	NO. of PAHs	Ref.
Harbin, Northeast (China)	4	Domestic/industrial	8200	16 PAHs	[32]
Guangzhou, (China)	10	Domestic/industrial	6386	16 PAHs	[31]
Taiwan (China)	4	Domestic	750	16 PAHs	[11]
Hong Kong, (China)	11	Domestic/industrial	30,000	16 PAHs	[9]
Beijing, (China)	12	Domestic/industrial	1551	15 PAHs	[29]
Paris, (France)	3	Domestic/industrial	2518	13 PAHs	[35]
Guangdong, (China)	6	Domestic/industrial	3467	15 PAHs	[33]
Korea	6	Domestic/industrial	10,400	16 PAHs	[30]
Guangdong, (China)	19	Domestic/industrial	1276	15 PAHs	[7]
Spanish Madrid	19	Domestic/industrial	5118	27 PAHs	[34]
Tunisian, Northern	9	Domestic/industrial	11,216	16 PAHs	[14]
Heilongjiang (China)	10	Domestic/industrial	2030	16 PAHs	This study
Heilongjiang (China)	10	Domestic/industrial	202	33 Me-PAHs	This study

**Table 3:** Toxic equivalency factors for PAHs in sludge (ng/g dw).

PAHs	TEF	W1	W2	W3	W4	W5	W6	W7	W8	W9	W10
NaP	0.001	0.29	0.27	0.22	0.09	0.16	0.009	0.21	0.19	0.64	0.37
Acy	0.001	0.05	0.04	0.02	0.01	0.02	0.005	0.01	0.01	0.03	0.02
Ace	0.001	0.03	0.04	0.02	0.01	0.03	0.008	0.01	0.02	0.05	0.04
Flu	0.001	0.13	0.15	0.06	0.04	0.09	0.03	0.04	0.08	0.19	0.19
Phe	0.001	0.43	0.67	0.18	0.27	0.67	0.12	0.22	0.30	0.48	0.52
Ant	0.01	0.36	0.61	0.24	0.21	0.85	0.14	0.13	0.37	0.48	0.29
Fluo	0.001	0.23	0.33	0.20	0.10	0.71	0.07	0.14	0.12	0.21	0.17
Pyr	0.001	0.26	0.35	0.20	0.12	0.57	0.07	0.19	0.11	0.19	0.15
BaA	0.1	6.31	9.69	4.10	3.14	23.0	1.89	4.57	3.04	6.28	3.08
Chr	0.01	0.83	1.05	1.47	0.57	3.54	0.33	0.60	1.18	1.07	1.04
BbF	0.1	11.5	17.6	22.1	11.4	38.5	5.31	11.2	9.64	17.2	13.0
BkF	0.1	3.54	4.26	4.29	2.87	9.96	1.22	2.92	2.74	3.94	3.24
BaP	1	69.3	98.0	96.7	44.2	225	27.4	67.0	28.0	78.1	31.8
IcdP	0.1	7.93	15.1	17.8	7.83	35.2	5.71	8.76	7.16	12.3	4.47
DahA	1	13.9	36	47.4	13.7	63.9	10.4	16.9	13.9	26.7	21.5
BghiP	0.01	1.05	1.43	2.15	0.87	3.25	0.61	1.18	0.71	1.39	0.18
Mean	0.15	7.26	11.6	12.3	5.34	25.3	3.34	7.13	4.23	9.34	5.01
Min	0.001	0.03	0.04	0.02	0.01	0.02	0.005	0.01	0.01	0.03	0.02
Max	1	69.3	98.0	96.7	44.2	225	27.4	67.0	28.0	78.1	31.8
∑ PAHs_carc_	2.42	113	181	194	83.8	399	52.3	112	66.7	145	78.2
∑ 16 PAHs	2.43	116	185	197	85.5	406	53.4	114	67.7	149	80.2

## Data Availability

The data collected are property of our research center but will be made available by the corresponding author when requested.

## Supplementary Materials

Electronic Supplementary Information is available for this manuscript.

## Sample Availability

Not available.
