# Supplementary material for: Determination of Polycyclic Aromatic Hydrocarbons and Their Methylated Derivatives in Sewage Sludge from Northeastern China: Occurrence, Profiles and Toxicity Evaluation
Source: Molecules. 2021 May 6;26(9):2739. doi: 10.3390/molecules26092739 (PMC8124507; doi:10.3390/molecules26092739)
Supplement: Supplementary file 1 [file molecules-26-02739-s001.zip › molecules-1164381-supplementary.pdf]

molecules

MDPI

Supplemental Information

Determination of Polycyclic Aromatic Hydrocarbons and their Methylated Derivatives in Sewage Sludge from Northeastern China: Occurrence, Profiles and Toxicity Evaluation

Rashid Mohammed [1,2,3], Zi-Feng Zhang [1,2,3,*], Ze Kan [4], Chao Jiang [4], Li-Yan Liu [1,2,3], Wan-Li Ma [1,2,3], Wei-Wei Song [1,2,3], Anatoly Nikolaev [5] and Yi-Fan Li [1,2,3,6,*]

[1] International Joint Research Center for Persistent Toxic Substances (IJRC-PTS), State Key Laboratory of Urban Water Resource and Environment, Harbin Institute of Technology (HIT), Harbin 150090, China; m13045102069@163.com (R.M.); llyan7664@163.com (L.-Y.L.); mawanli002@163.com (W.-L.M.); weiweiwendysong@126.com (W.-W.S.)
[2] International Joint Research Center for Arctic Environment and Ecosystem (IJRC-AEE), Polar Academy/School of Environment, Harbin Institute of Technology (HIT), Harbin 150090, China
[3] Heilongjiang Provincial Key Laboratory of Polar Environment and Ecosystem (HPKL-PEE), Harbin Institute of Technology (HIT), Harbin 150090, China
[4] Heilongjiang Institute of Labor Hygiene and Occupational Diseases, Harbin 150028, China; yuanban888@126.com (Z.K.); sey53969319@126.com (C.J.)
[5] Institute of Natural Sciences, North-Eastern Federal University, 677000 Yakutsk, Russia; an.nikolaev@s-vfu.ru
[6] IJRC-PTS-NA, Toronto, Ontario, M2N 6X9, Canada
* Correspondence: zhangzifeng@hit.edu.cn (Z.-F.Z.); dr_li_yifan@163.com (Y.-F.L.); Tel.: +86-451-8628-9130

Table S1. Basic information about the study WWTPs of PAHs and Me-PAHs in sewage sludge.

Site	longitude	latitude	Start Time	type of treatment	Design capacity (m^3/d)	Source
**W1**	123	47.2	2003	Activated Sludge Process	61,600	Domestic and industrial
**W2**	129	44.6	2003	Anoxic/Oxic Process	100,000	Domestic and industrial
**W3**	130	46.8	2006	Sequencing Batch Reactors Process	60,000	Domestic and industrial
**W4**	130	46.8	2010	Sequencing Batch Reactors Process	44,000	Domestic
**W5**	126	45.7	2011	Anoxic/Oxic Process	38,000	Domestic
**W6**	126	45.5	2009	Anoxic/Oxic Process	64,000	Domestic and industrial
**W7**	126	45.6	2013	Cyclic Activated Sludge System	46,000	Domestic
**W8**	126	45.8	2005	Anoxic/Oxic Process	286,400	Domestic and industrial
**W9**	126	45.8	2003	Anoxic/Oxic Process	143,700	Domestic and industrial
**W10**	126	45.9	2011	Cyclic Activated Sludge System	100,100	Domestic and industrial

Table S2. GC-MS/MS detection parameters of PAHs, including the optimized retention time, transitions and collision energy (CE).

Analytes	Retention Time (min)	Transition 1 (m/z)	CE 1 (eV)	Transition 2 (m/z)	CE 2 (eV)
Naphthalene	5.077	128→102	20	128→127	20
Acenaphthylene	7.142	152→150	25	152→151	25
Acenaphthene	7.399	153→152	25	153→151	25
Fluorene	8.213	166→165	25	165→163	30
Phenanthrene	10.630	178→176	25	178→152	25
Anthracene	10.793	178→176	25	178→152	25
Fluoranthene	15.356	202→200	35	202→201	25
Pyrene	16.053	202→200	35	202→201	25
Benz(a)anthracene	19.670	228→226	30	228→202	30
Chrysene	19.779	228→226	30	228→202	30
Benzo[b]fluoranthene	22.325	252→250	30	252→226	25
Benzo[k]fluoranthene	22.389	252→250	30	252→226	25

Benzo[a]pyrene	22.996	252→250	30	252→226	25
Indeno[1,2,3-cd]pyrene	25.331	276→274	45	276→272	50
Dibenz(a,h)anthracene	25.410	278→274	55	278→276	50
Benzo[g,h,i]perylene	25.914	276→274	45	276→272	50
**Me-PAHs**					
2-Methylnaphthalene	9.245	141→115	20	142→141	20
1-Methylnaphthalene	9.548	141→115	20	142→141	20
2,6-Dimethylnaphthalene	11.156	156→141	20	156→115	40
2,7-Dimethylnaphthalene	11.165	156→141	15	141→115	20
1,3-Dimethylnaphthalene	11.410	141→115	20	156→141	20
1,6-Dimethylnaphthalene	11.459	156→141	20	141→115	20
1,4-Dimethylnaphthalene	11.732	141→115	20	156→141	20
1,5-Dimethylnaphthalene	11.783	141→115	20	156→141	20
1,2-Dimethylnaphthalene	11.982	141→115	25	156→141	15
2-Methylphenanthrene	19.414	192→191	20	191→189	30
2-Methylanthracene	19.680	192→191	20	192→189	40
1-Methylanthracene	19.964	192→191	20	192→189	40
1-Methylphenanthrene	20.029	192→191	20	191→189	30
9-Methylanthracene	21.015	192→191	20	191→189	30
3,6-Dimethylphenanthrene	22.588	206→191	20	206→189	45
2,3-Dimethylanthracene	25.113	206→191	20	206→189	45
9,10-Dimethylanthracene	27.022	206→191	20	191→189	30
9-Methyl-9-phenylfluorene	27.197	241→239	35	256→241	15
2-Methylfluoranthene	28.001	216→215	25	215→213	40
1-Methylpyrene	29.192	216→215	25	215→189	30
1,2-Methylbenz[a]anthracene	31.975	242→241	20	242→239	50
7,9-Methylbenz[a]anthracene	32.038	242→241	20	242→239	45
4,6-Methylbenz[a]anthracene	32.100	242→241	20	242→239	45
3,5-Methylbenz[a]anthracene	32.218	242→241	20	242→239	50
10-Methylbenz[a]anthracene	32.453	242→241	20	242→239	45
5,8-Dimethylbenzo[c]phenanthrene	32.751	256→241	20	256→239	50
6,8-Dimethylbenz[a]anthracene	32.848	256→239	45	256→241	20
3,9-Dimethylbenz[a]anthracene	32.891	256→239	50	256→255	25
7,12-Dimethylbenz(a)anthracene	33.305	256→241	20	256→239	50
3-Methylcholanthrene	34.467	268→252	40	268→253	20
8,9-Methylbenzo[a]pyrene	34.512	266→265	20	266→263	50
7,10-Methylbenzo[a]pyrene	34.693	266→265	20	266→263	50
7,10-Dimethylbenzo[a]pyrene	35.722	280→265	20	280→264	35

Table S3. Concentration of PAHs and Me-PAHs in sewage sludge from 10 WWTP in Northeast of China.

Chemicals	Ring	Mean+SD	Median	Minimum	Maximum	Skewness	Kurtosis
PAHs							
NaP	2	250 ±170	221	9.38	641	1.21	2.71
Acy	2	25.0±15.4	21.4	5.86	55.8	0.99	0.47
Ace	3	30.2±15.2	31	8.51	54.5	0.11	1.18
Flu	3	104 ±60.3	88.2	32	197	0.49	1.24
Phe	3	391 ±198	367	129	677	0.28	1.30
Ant	3	37.3±22.3	33	13.4	85.1	1.15	1.08
Fluo	4	233 ±185	193	77.3	716	2.31	5.95
Pyr	4	225 ±148	192	73.9	577	1.68	3.14
BaA	4	65.1±62.3	43.3	18.9	230	2.47	6.60
Chr	4	117 ±89.7	104	33.9	354	2.37	6.55
BbF	5	157 ±92.7	122	53.1	385	1.80	4.02
BkF	5	39.0±23.1	33.9	12.2	99.6	2.24	6.40

BaP	5	76.6±58.7	68.1	27.4	225	2.03	5.00
IcdP	6	122 ±91.2	83.5	44.7	352	2.07	4.78
DahA	5	26.4±17.5	19.2	10.4	63.9	1.32	0.96
BghiP	6	128 ±87.4	111	18	325	1.31	2.13
∑PAHs		203 ±133	1730	567	5030	23.8	46.1
Me-PAHs							
2-MNAP	2	25.1±13.9	15.2	1.13	25.1	0.46	1.88
1-MNAP	2	17.2±9.28	9.94	0.59	17.3	0.32	1.98
2,6-DMNAP	2	17.4±8.98	7.24	2.29	17.4	0.64	1.17
2,7-DMNAP	2	17.4±8.98	7.24	2.29	17.4	0.64	1.17
1,3-DMNAP	2	18.5±8.28	8.21	1.11	18.5	0.93	2.27
1,6-DMNAP	2	21.4±8.92	7.85	3.85	21.5	1.96	4.54
1,4-DMNAP	2	8.53±3.53	3.45	0.61	8.53	1.42	3.61
1,5-DMNAP	2	6.25±2.32	1.95	1.06	6.25	2.43	6.81
1,2-DMNAP	2	5.31±2.43	2.06	0.38	5.31	0.98	0.76
2-MPHE	3	17.1±5.91	3.71	1.3	17.2	1.36	0.79
2-MANT	3	24.6±8.70	5.90	2.46	24.6	1.51	1.40
1-MANT	3	18.0±6.47	4.17	1.36	18.1	1.34	0.53
1-MPHE	3	14.3±5.16	3.88	0.91	14.3	1.39	1.11
9-MANT	3	90.6±28.6	19.6	3.36	90.6	1.47	1.40
3,6-DMPHE	3	7.34±3.12	2.16	0.69	7.34	1.08	0.04
2,3-DMA	3	3.34±1.17	0.96	0.28	3.34	1.58	2.66
9,10-DMA	3	2.65±0.64	0.24	0.08	2.65	1.76	1.80
9-MMHEN	3	0.03±0.01	BDL	BDL	0.03	0.58	1.54
2-MFLU	4	7.43±2.60	1.70	0.82	7.43	1.69	1.83
1-MPYR	4	9.41±4.51	3.83	1.03	9.41	0.59	0.68
1,2-MBaA	4	2.53±1.19	1.07	0.25	2.53	0.68	0.69
7,9-MBaA	4	24.1±12.3	11.1	3.1	24.1	0.26	1.33
4,6-MBaA	4	7.15±3.62	3.20	0.92	7.15	0.50	1.24
3,5-MBaA	4	14.0±8.93	7.98	5.84	14.0	1.17	0.90
10-MBaA	4	9.97±6.15	5.59	3.56	9.97	0.81	0.79
5,8-DMBcPH	4	24.4±11.6	10.5	2.81	24.4	0.49	0.96
6,8-DMBaA	4	10.2±4.93	4.52	1.05	10.2	0.27	1.21
3,9-DMBaA	4	14.5±7.46	6.77	1.68	14.5	0.25	1.42
7,12-DMBaA	4	5.13±2.51	2.56	0.71	5.13	0.29	1.00
3-MCHA	3	1.52±0.35	BDL	BDL	1.52	1.38	0.26
3,9-MCHA	3	22.7±9.39	7.47	2.28	22.7	0.85	0.22
7,10-MBaP	5	5.72±2.23	1.65	0.35	5.72	0.72	0.73
7,10-DMBaP	5	5.24±1.38	0.88	BDL	5.24	1.68	2.94
∑Me-PAHs		205 ±139	172	48.1	479	32.0	24.9

Table S4. Fluxes calculations of PAHs and Me-PAHs (g/d dw) from sewage sludge in Northeast of China.

**Chemicals**	**Sampling Sites**									
**PAHs**	**W1**	**W2**	**W3**	**W4**	**W5**	**W6**	**W7**	**W8**	W9	W10
NaP	9.31	12.7	9.85	4.47	11.5	0.88	11.3	2.75	10.2	6.73
Acy	1.78	2.07	0.98	0.59	1.39	0.55	0.87	0.18	0.48	0.49
Ace	1.20	1.89	1.16	0.61	2.42	0.79	0.91	0.28	0.87	0.84
Flu	4.25	6.93	2.99	2.12	6.34	3.01	2.57	1.16	3.07	3.55
Phe	13.7	31.1	7.99	12.7	46.0	12.1	11.7	4.27	7.82	9.46
Ant	1.17	2.80	1.09	1.00	5.78	1.40	0.70	0.52	0.77	0.52
Fluo	7.47	15.3	9.21	4.85	48.7	7.26	7.32	1.74	3.50	3.19
Pyr	8.43	16.5	9.21	5.72	39.3	6.94	10.0	1.54	3.07	2.73
BaA	2.02	4.45	1.80	1.44	15.6	1.78	2.37	0.42	1.00	0.55
Chr	2.68	4.83	6.50	2.66	24.1	3.19	3.15	1.66	1.72	1.88
BbF	3.69	8.09	9.72	5.26	26.1	4.99	5.85	1.34	2.75	2.34
BkF	1.13	1.96	1.88	1.32	6.77	1.15	1.52	0.38	0.63	0.58

BaP	2.21	4.50	4.25	2.03	15.3	2.57	3.48	0.39	1.25	0.57
IcdP	2.53	6.94	7.85	3.60	23.9	5.37	4.55	1.00	1.97	0.80
DahA	0.44	1.65	2.08	0.63	4.34	0.98	0.87	0.19	0.42	0.38
BghiP	3.37	6.62	9.46	4.01	22.1	5.77	6.14	1.00	2.23	0.32
Min	0.44	1.65	0.98	0.59	1.39	0.55	0.70	0.18	0.42	0.32
Max	13.7	31.1	9.85	12.7	48.7	12.1	11.7	4.27	10.2	9.46
Mean	4.09	8.03	5.38	3.32	18.7	3.67	4.59	1.18	2.61	2.18
∑PAHs	65.5	128	86.1	53.1	300	58.8	73.5	18.8	41.8	34.9
Me-PAHs										
2-MNAP	9.19	16.2	15.7	9.52	25.1	16.3	15.2	15.7	14.7	1.12
1-MNAP	6.22	10.3	10.9	6.32	17.2	9.98	11.3	9.86	9.89	0.58
2,6-DMNAP	7.12	17.4	6.42	4.53	15.2	8.68	7.37	3.50	2.29	17.2
2,7-DMNAP	7.12	17.4	6.42	4.53	15.2	8.68	7.37	3.50	2.29	17.2
1,3-DMNAP	10.3	11.2	8.28	5.60	18.5	8.65	8.13	6.04	4.92	1.11
1,6-DMNAP	8.19	12.3	7.52	5.31	21.4	8.51	9.77	5.86	3.85	6.47
1,4-DMNAP	4.50	4.13	3.63	2.44	8.52	3.27	3.73	2.51	1.99	0.60
1,5-DMNAP	2.47	2.07	2.23	1.83	6.24	1.78	2.71	1.62	1.05	1.16
1,2-DMNAP	4.51	2.64	2.01	1.72	5.30	2.11	2.51	1.77	1.40	0.37
2-MPHE	2.63	9.47	4.01	2.32	17.1	4.27	3.40	1.92	1.29	12.6
2-MANT	3.57	11.4	6.15	3.72	24.6	6.92	5.64	4.04	2.46	18.4
1-MANT	2.48	9.24	4.13	2.51	18.0	5.00	4.21	2.63	1.36	15.0
1-MPHE	1.48	5.11	4.02	2.55	14.2	5.67	3.74	2.43	0.91	11.3
9-MANT	3.35	35.2	24.5	14.6	90.6	24.8	5.18	10.3	10.7	66.8
3,6-DMPHE	1.91	4.47	2.75	2.17	7.33	2.05	2.15	1.08	0.68	6.62
2,3-DMA	0.45	1.57	1.09	0.60	3.34	0.92	1.00	0.45	0.27	1.98
9,10-DMA	0.08	0.33	0.34	0.08	0.15	0.07	0.11	2.64	2.07	0.52
9-MMHEN	0.02	0.03	BDL	BDL	BDL	0.02	BDL	0.02	0.01	BDL
2-MFLU	1.21	1.65	2.83	1.53	7.42	1.82	1.75	1.26	0.81	5.71
1-MPYR	2.00	5.82	4.10	3.31	9.41	5.77	3.56	1.49	1.02	8.58
1,2-MBaA	0.40	1.16	1.42	0.61	2.53	1.59	0.99	0.52	0.24	2.47
7,9-MBaA	4.75	12.6	19.7	9.68	18.6	17.1	8.57	5.31	3.10	24.1
4,6-MBaA	1.38	3.26	6.48	3.14	6.02	3.90	2.28	1.63	0.92	7.14
3,5-MBaA	5.84	7.70	10.1	7.98	8.65	12.0	7.60	7.98	7.39	14.0
10-MBaA	3.55	5.55	7.55	5.64	7.03	7.34	4.99	5.28	4.58	9.97
5,8-DMBcPH	4.15	12.6	16.5	8.50	14.5	24.4	7.33	4.52	2.80	20.9
6,8-DMBaA	1.39	5.39	8.08	3.64	7.32	7.04	3.37	1.79	1.05	10.2
3,9-DMBaA	2.74	7.41	12.0	6.14	10.1	12.0	4.84	3.12	1.68	14.5
7,12-DMBaA	0.75	3.98	3.30	2.00	3.12	5.13	1.66	1.05	0.71	3.44
3-MCHA	BDL	BDL	BDL	BDL	BDL	BDL	BDL	1.51	0.70	1.33
3,9-MCHA	2.28	10.5	16.6	6.28	15.3	8.66	4.81	4.18	2.39	22.7
7,10-MBaP	0.37	2.90	3.78	1.13	4.35	2.16	0.92	0.60	0.34	5.72
7,10-DMBaP	0.35	0.30	0.94	1.45	2.85	1.90	0.81	BDL	BDL	5.24
Min	BDL	BDL	BDL	BDL	BDL	BDL	BDL	BDL	BDL	BDL
Max	10.3	35.2	24.5	14.6	90.6	24.8	15.2	15.7	14.7	66.8
Mean	3.24	7.63	6.78	3.98	12.9	6.93	4.46	3.52	2.72	10.1
∑Me-PAHs	106	251	223	131	425	228	147	116	90.0	335

Table S5. Factor pattern for total PAHs (a) and Me-PAHs (b) in sewage sludge in Northeast of China.

	PAHs	PC1	PC2
a:	NaP	0.18	**0.85**
	Acy	0.28	**0.71**
	Ace	0.49	**0.85**
	Flu	0.26	**0.94**
	Phe	0.74	**0.53**
	Ant	**0.91**	0.18

	Fluo	**0.98**	-0.07	
	Pyr	**0.96**	-0.03	
	BaA	**0.96**	-0.13	
	Chr	**0.93**	-0.19	
	BbF	**0.96**	-0.14	
	BkF	**0.98**	-0.12	
	BaP	**0.96**	-0.20	
	IcdP	**0.94**	-0.29	
	DahA	**0.90**	-0.16	
	BghiP	**0.87**	-0.33	
	Explained variance (%)	67.0	22.0	
b:	Me-PAHs	PC1	PC2	PC3
	2-MNAP	0.09	**0.80**	**0.49**
	1-MNAP	0.09	**0.82**	**0.40**
	2,6-DMNAP	**0.84**	0.04	-0.05
	2,7-DMNAP	**0.84**	0.04	-0.05
	1,3-DMNAP	0.33	**0.92**	0.06
	1,6-DMNAP	0.65	0.74	0.03
	1,4-DMNAP	0.36	**0.91**	-0.00
	1,5-DMNAP	0.52	**0.80**	-0.09
	1,2-DMNAP	0.16	**0.90**	-0.19
	2-MPHE	**0.92**	0.22	-0.01
	2-MANT	**0.93**	0.19	0.05
	1-MANT	**0.94**	0.13	-0.01
	1-MPHE	**0.95**	0.10	0.01
	9-MANT	**0.93**	0.12	0.06
	3,6-DMPHE	**0.94**	0.10	-0.13
	2,3-DMA	**0.92**	0.33	-
	9,10-DMA	0.47	-0.17	**0.59**
	9-MMHEN	0.32	0.13	**0.45**
	2-MFLU	**0.91**	0.11	-0.08
	1-MPYR	**0.98**	0.03	-0.02
	1,2-MBaA	**0.98**	-0.05	0.05
	7,9-MBaA	**0.91**	-0.29	0.04
	4,6-MBaA	**0.89**	-0.24	-0.01
	3,5-MBaA	0.65	-0.67	0.19
	10-MBaA	**0.81**	-0.53	0.14
	5,8-DMBcPH	0.77	-0.33	0.19
	6,8-DMBaA	**0.90**	-0.32	0.06
	3,9-DMBaA	**0.85**	-0.36	0.08
	7,12-DMBaA	0.70	-0.16	0.29
	3-MCHA	0.06	-0.55	0.35
	3,9-MCHA	**0.89**	-0.33	0.02
	7,10-MBaP	**0.94**	-0.23	0.03
	7,10-DMBaP	**0.82**	-0.40	-0.23
	Explained variance (%)	58.8	22.0	4.37

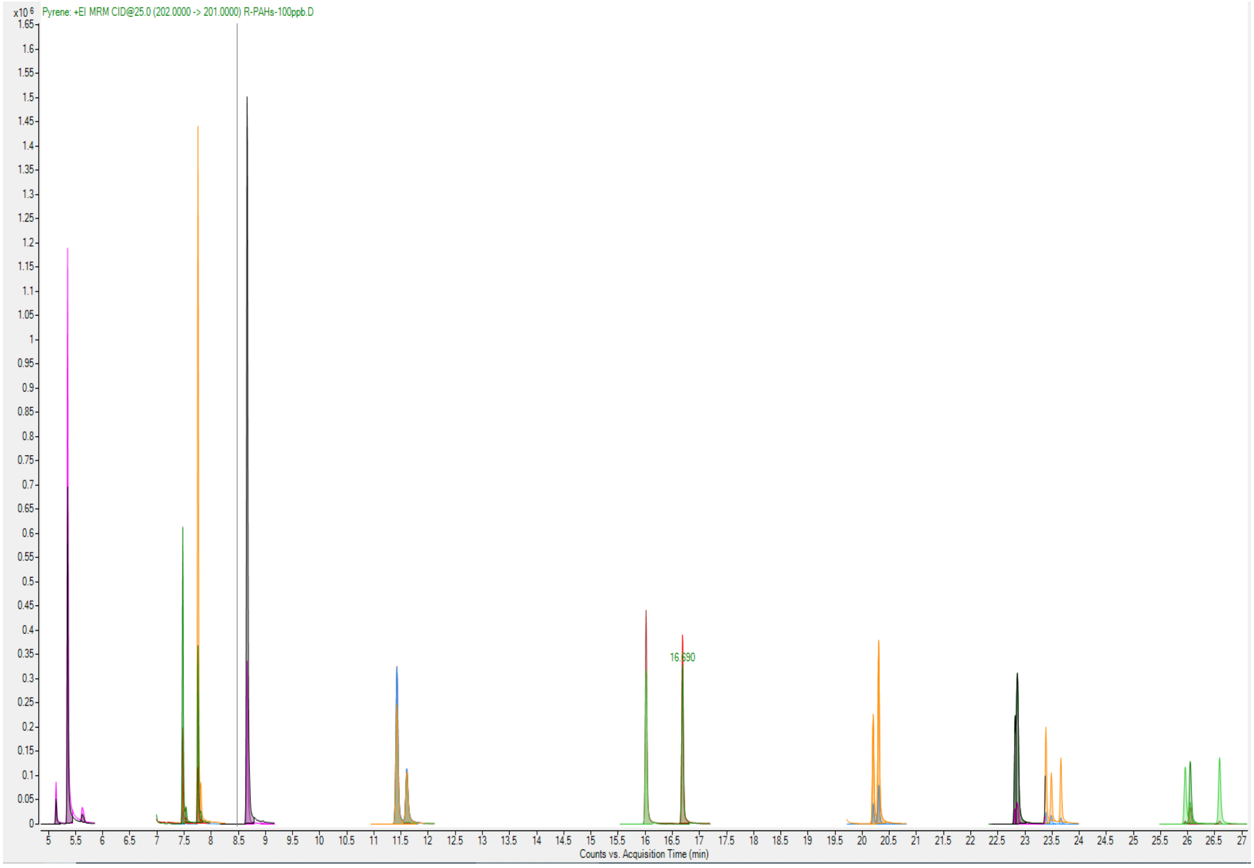

Figure S1. MRM chromatogram of target PAHs.

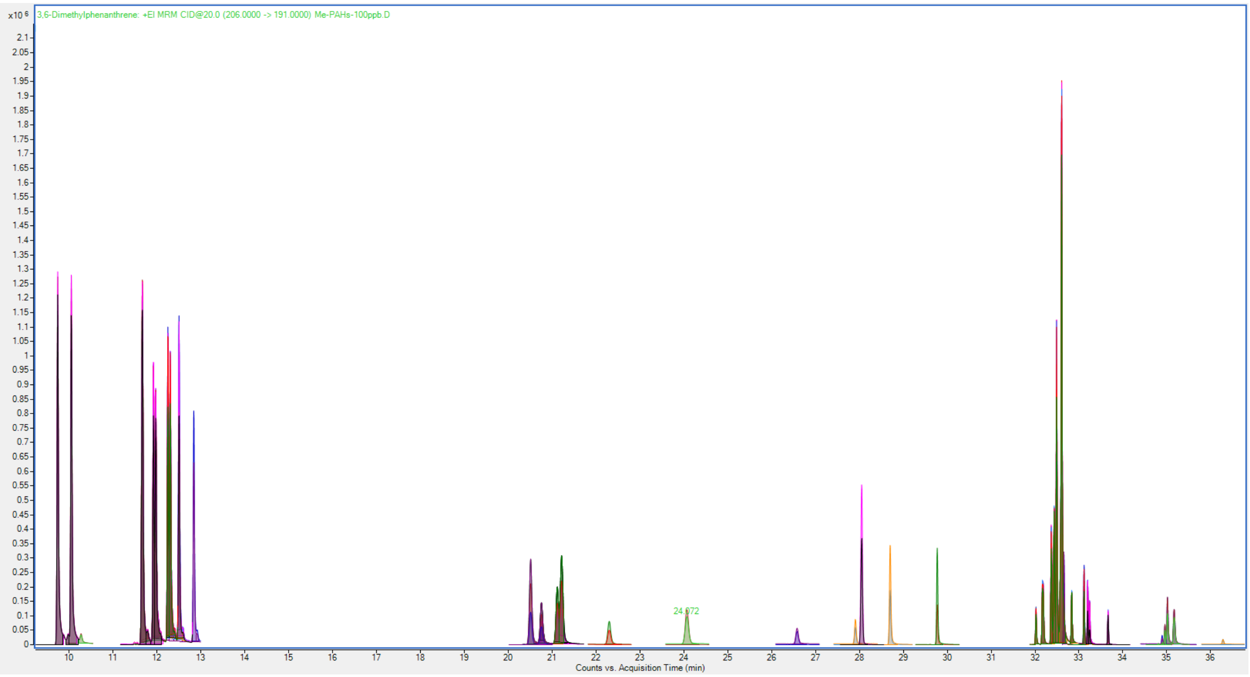

Figure S2. MRM chromatogram of target Me-PAHs.

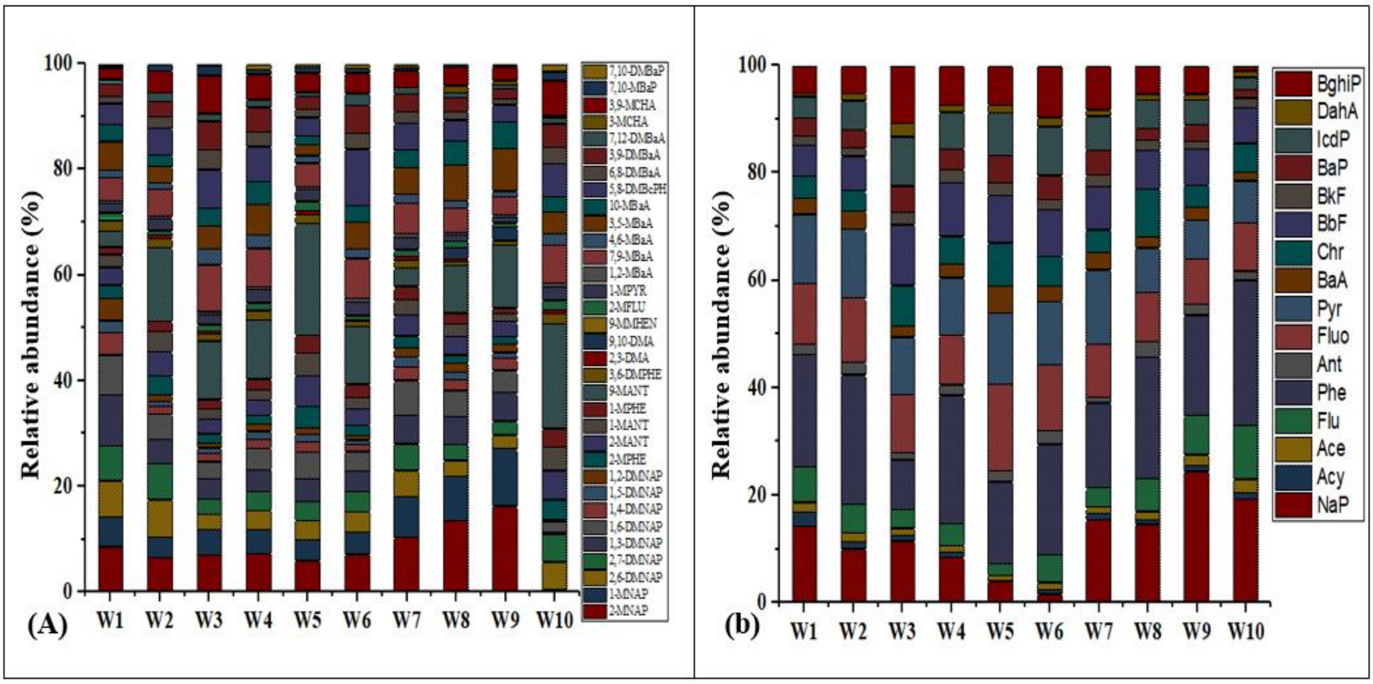

Figure S3. Average composition of individual Me-PAHs (a) and PAHs (b) in sludge samples.

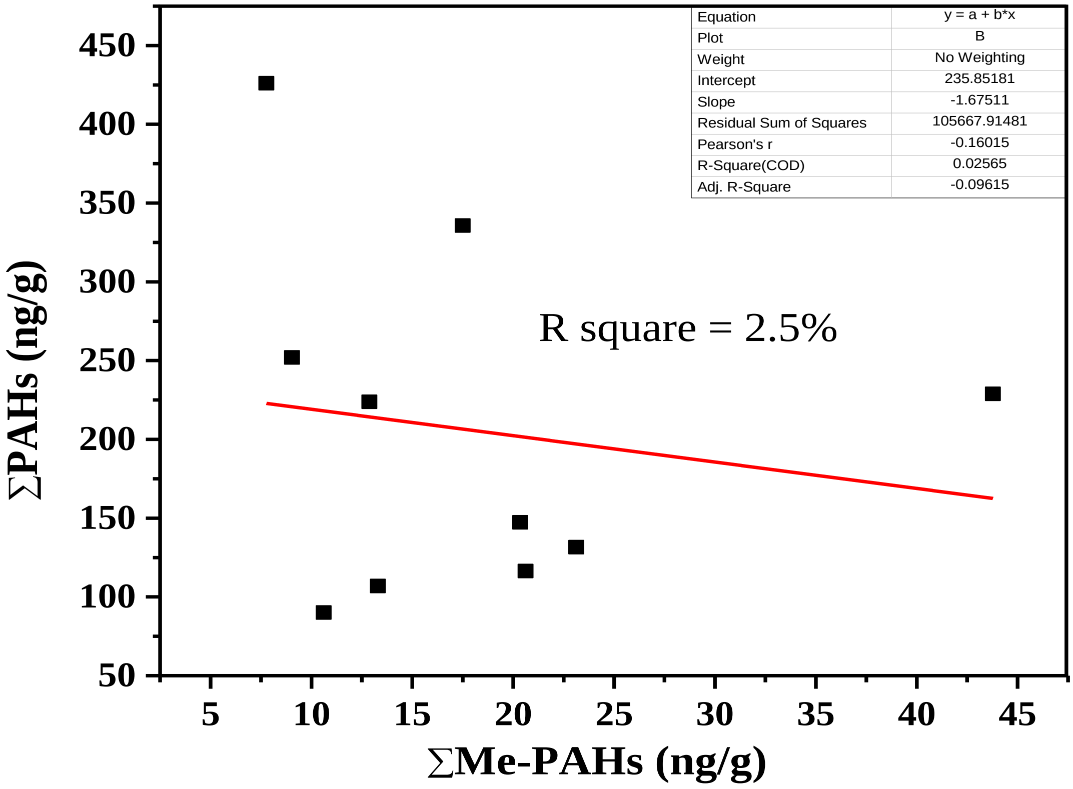

Figure S4. Correlation analysis between PAHs and Me-PAHs in sludge samples.
